# Linking changes in individual specialization and population niche of space use across seasons in the great evening bat (*Ia io*)

**DOI:** 10.1186/s40462-023-00394-1

**Published:** 2023-06-07

**Authors:** Zhiqiang Wang, Lixin Gong, Zhenglanyi Huang, Yang Geng, Wenjun Zhang, Man Si, Hui Wu, Jiang Feng, Tinglei Jiang

**Affiliations:** 1 Jilin Provincial Key Laboratory of Animal Resource Conservation and Utilization, Northeast Normal University, 2555 Jingyue Street, Changchun, 130117 China; 2 Key Laboratory of Vegetation Ecology of Education Ministry, Institute of Grassland Science, Northeast Normal University, 2555 Jingyue Street, Changchun, 130117 China; 3 College of Life Science, Jilin Agricultural University, 2888 Xincheng Street, Changchun, 130118 China

**Keywords:** Bats, Individual specialization, Niche evolution, Resource changes, Spatial ecology

## Abstract

**Background:**

The niche breadth of an animal population comprises both within-individual and between-individual variation (individual specialization). Both components can be used to explain changes in population niche breadth, and this has been extensively investigated in dietary niche dimension studies. However, little is known about how changes in food resources or environmental factors across seasons affect changes in individual and population space use within the same population.

**Methods:**

In this study, we used micro-GPS loggers to capture the space use of individuals and of a population of the great evening bat (*Ia io*) in summer and autumn. We used *I. io* as a model to investigate how individual spatial niche breadth and spatial individual specialization affect changes in population niche breadth (home range and core area sizes) across seasons. Additionally, we explored the drivers of individual spatial specialization.

**Results:**

We found that the population home range and the core area of *I. io* did not increase in autumn when insect resources were reduced. Moreover, *I. io* showed different specialization strategies in the two seasons: higher spatial individual specialization in summer and lower individual specialization but broader individual niche breadth in autumn. This trade-off may maintain the dynamic stability of the population spatial niche breadth across seasons and facilitate the population response to changes in food resources and environmental factors.

**Conclusions:**

Like diet, spatial niche breadth of a population also may be determined by a combination of individual niche breadth and individual specialization. Our work provides new insights into the evolution of niche breadth from the spatial dimension.

**Supplementary Information:**

The online version contains supplementary material available at 10.1186/s40462-023-00394-1.

## Background

Studies of niche breadth can help us to understand patterns of biological adaptation, species formation, and range variation; additionally, changes in niche breadth can influence how species respond to changes in climate, environment, and resources [1]. The niche breadth of an animal population comprises both within-individual and between-individual variation (individual specialization) [1, 2]. In this case, changes in population niche breadth usually derive from individual niche breadth and/or individual specialization. When the available food resources decrease, the individual niche breadth should be expected to increase, and the population niche breadth will expand accordingly [3, 4]. For example, the increase in dietary niche breadth of a moose population was due to an increase in the individual dietary niche breadth rather than an increase in among-individual variation [5]. The alternative hypothesis suggests that increased inter-individual variation in turn increases the niche breadth of the population [2, 6]. For example, in seal populations, the greater the degree of individual specialization, the greater the population niche breadth [7]. Previous studies mainly focused on linking the changes between individual-level niche and population-level niche to the dietary niche dimension [6, 8–11]. Although there has been considerable research examining changes in niche breadth, relatively few studies have focused specifically on the spatial dimension of these changes. Neglecting this dimension can impede efforts to test the universality of evolutionary mechanisms driving changes in niche breadth, highlighting the need for further investigation in this area. Additionally, individual specialization has been demonstrated in animal taxa such as gastropods, crustaceans, fish, reptiles, birds, and mammals [12–14]. However, these studies have focused on individual specialization in the utilization of food resources and have demonstrated the effect on population dietary niche breadth. In contrast, little is known about the patterns of individual specialization in space use or the effects on the spatial population niche. In sum, testing the relationships between spatial individual specialization and population niche of space use would not only contribute to increasing knowledge in the field of movement ecology, but would also be helpful for expanding the application range of the relevant ecological hypothesis or theory [i.e., niche variation hypothesis (NVH) or optimal foraging theory (OFT)].

The search for resources, especially for food, is one of the most important drivers of animal movement and is affected by an animal’s condition, as well as by resource and landscape diversity [15]. Recently, movement ecology has received increasing attention following the proposal of a conceptual framework [16]. Like food resources, space can be considered a resource in ecological theory [16, 17]. In general, animal home ranges, i.e., the niche breadth in terms of spatial use, increase as resource diversity decreases. During the dry season, when available food resources are decreased, many vertebrate populations expand their home ranges to obtain the resources they need to survive; for example, the Guatemalan beaded lizard (*Heloderma charlesbogerti*) [18], the blue-eyed black lemur (*Eulemur flavifrons*) [19], the African straw-colored fruit bat (*Eidolon helvum*) [20], and the roe deer (*Capreolus capreolus*) [21]. Core areas are commonly defined as the geographic regions most frequently visited and utilized by individuals, often indicating the location of their home site, refuges, or important food sources [22, 23]. These areas are typically characterized by the highest intensity of use or the greatest density of location points. However, existing studies have largely focused on the dynamics of spatial use at the animal population level (mean home ranges), while the patterns of spatial use at the individual level and their effects on the spatial niche of the population remain unclear.

Recently, with the increase in the availability of individual movement and distribution data via advanced tracking devices and analytical tools [24, 25], researchers have become aware of differences in spatial use of individuals and have emphasized the importance of studying individual specialization in regard to space. Spatial specialization is currently primarily studied in seabirds and fish, a result that may be related to their strong dispersal ability and continuous movement during specific life stages [16]. This individual specialization is usually measured in terms of environmental preferences, differences in activity patterns, fidelity to foraging sites, and repeatability of habitat selection [26–28]. Although spatial specialization strategies are considered more appropriate in areas with heterogeneous environments, high resource predictability, and among individuals with more daring personalities [17, 28–30], how individuals adjust spatial specialization in response to environmental changes remains largely unexplored. Thus, it is unclear which factors drive space individual specialization. Directly exploring the level of specialization in spatial use by considering the volume of spatial niches and the overlap of individual spatial use opens new perspectives for studying individual specialization [17]. Recently, researchers have begun to examine relationships between individual spatial specialization and diet [17, 31]. However, to our knowledge, little is known about variation in spatial niche at both population and individual levels in the context of food resource decreases due to seasonality.

**Fig. 1:**
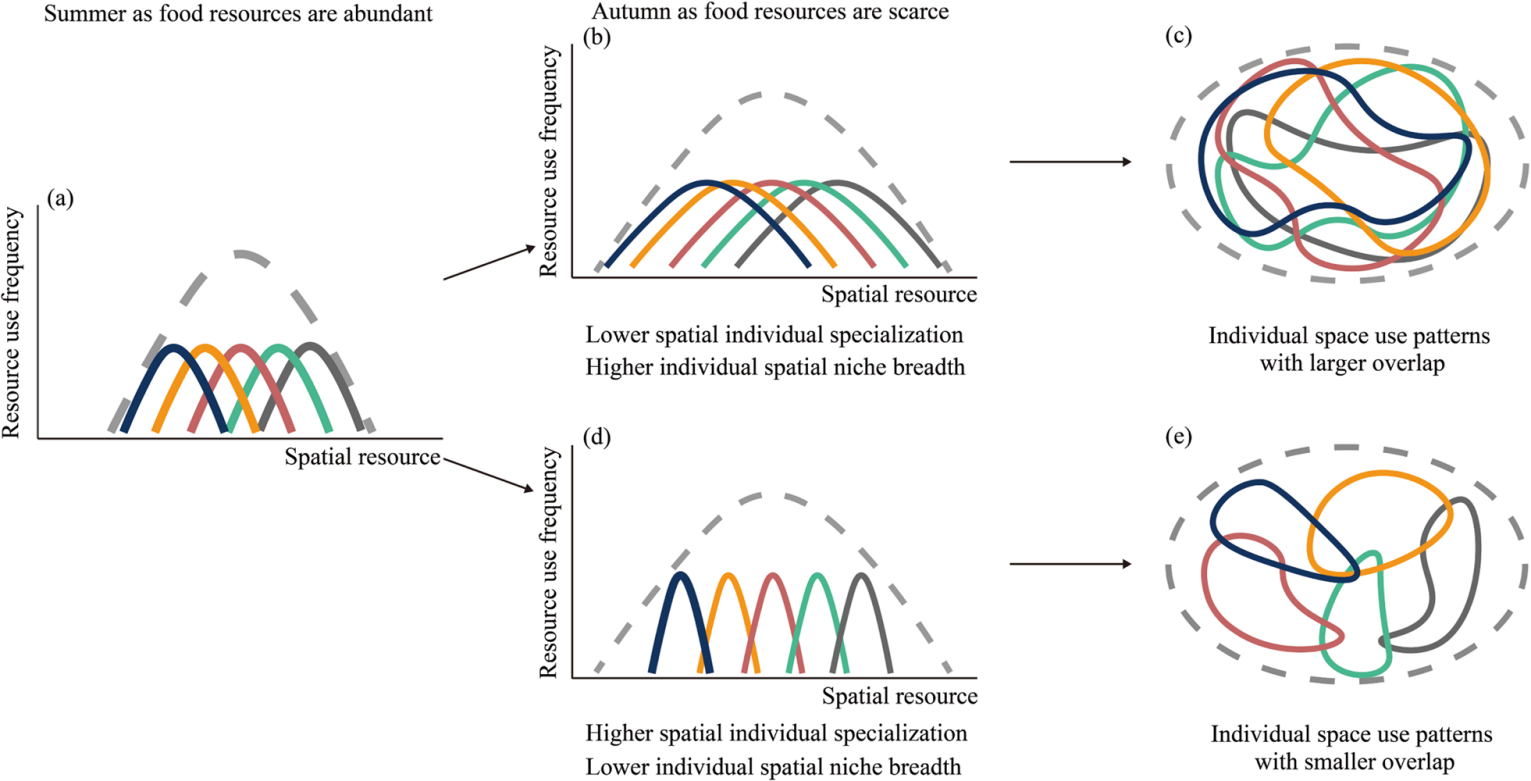
Conceptual framework linking individual specialization to population spatial niche breadth (adapted from Kerches-Rogeri et al.). **a** The population spatial niche breadth (dotted line) in summer when food resources are abundant. **b** The increase in population spatial niche breadth (dotted line) in autumn (when food resources are scarce) due to increases in individual spatial niche breadth (colored lines). That is, lower spatial individual specialization would be observed. **c** The hypothetical home range used by the population (dotted line) and individual bats (colored lines). **b** and **c** Generalist strategies in space use that would be selected in autumn. **d** The increase in population spatial niche breadth (dotted line) in autumn when food resources are scarce due to increases in spatial individual specialization (colored lines). That is, lower individual spatial niche breadth would be observed. **e** A hypothetical home range used by the population (dotted line) and individual bats (colored lines). **d** and **e** Specialist strategies in space use would be favored in autumn

As the only flying mammals, bats are excellent models for studying changes in population and individual niches under the framework of movement ecology, as well as for studying responses and adaptations to environmental changes in terms of spatial use [32]. The great evening bat (*Ia io*) hunts for insects in summer but it is known to prey upon nocturnally migrating birds in autumn when insect resources decrease [33–35]. Our previous study showed that significant changes in population and individual dietary niches of *I. io* were observed in summer when insect resources were abundant and in autumn when they became relatively scarce [36]. Thus, here we used miniature global positioning system (GPS) loggers to investigate changes in the spatial use of individuals and the population in *I. io* across seasons (summer and autumn). We tested the following hypotheses: first, we hypothesized that the spatial niche breadth (home range and core area sizes) of the *I. io* population would increase in autumn when food resources decreased compared to summer when food resources were abundant (Fig. 1a, b, d). Second, if the first hypothesis held, we hypothesized that the increase in the spatial niche breadth of the *I. io* population in autumn would stem from increases in individual spatial niche breadth (the range of spatial areas used by individuals) (Fig. 1a–c) or from increases in individual spatial specialization (less overlapping of spatial areas used by individuals) (Fig. 1a, d, and e). Third, if the spatial niche breadth of the *I. io* population was unchanged, we hypothesized that the spatial niche breadth and spatial individual specialization would vary between individuals of the same population in response to food and environmental resource variation across seasons. We predicted that the spatial niche breadth will be narrower and the degree of spatial individual specialization will be higher in summer than in autumn. Finally, because previous studies showed that individual spatial specialization may be affected by phenotypic traits (i.e., body mass), landscape characteristics (i.e., land use type, distance from roads, and village density), and food resources, we hypothesized that individual spatial specialization in *I. io* may be affected by factors such as body mass, insect resource diversity, and landscape variables.

## Methods

### Study area and species

The study area was located in the mountain region of southwestern China (Xingyi City, Guizhou Province). At this site the elevation ranges from 700 to 2200 m, and the topography is typical of the Castell landscape. The area is on one of the eight global migratory routes of birds in Central Asia (www.birdlife.org/worldwide/programme-additional-info/migratorybirds-and-flyways). There are many caves distributed in the area, among which the Feilong Cave (24° 58.41′ N, 104° 52.79′ E) is inhabited by the largest population of *I. io* (about 80) in China. Feilong Cave is mainly a colony of *I. io* males. Our previous study showed that *I. io* in this population mainly prey on insects in summer (June to August) and birds in autumn (September to November) [33]. In this study, fourteen adult male *I. io*, seven in November 2020 and seven in June 2021, were captured at Feilong Cave using mist nets. The population of *I. io* frequently changes its habitat, and it is generally believed that under natural conditions in the wild, individual movement and foraging patterns are independent, and that individuals of the same period may have similar movement patterns [37, 38]. As the number of individuals, we were able to capture in a short period of time was limited, our available sample size was restricted. However, we believe that our findings can still reflect the movement and spatial use of the population during the two seasons under study. The body mass of each bat was weighed using an electronic balance (DINING DH-I200, accuracy 0.01 g), and the forearm length was measured using digital calipers (TESA-CAL IP67, Switzerland, accuracy 0.01 mm).

### GPS tracking

A section of the hair between the shoulder blades of each bat was cut off using dissecting scissors. Then, a GPS device (HQXS, HQBG0603) with a GSM system (Global System for Mobile Communication, MC20, Quectel Wireless Solutions Co., Ltd., Shanghai, China) was glued to the back of the bat using skin glue (Medical-Surgical Glue, China). The GPS logger was fixed by hand for 60 s to avoid shifting and shedding. After the glue had fully dried, the bat was placed in a cloth bag and acclimated for 30 min to allow the logger to fit completely on the bat. All of the above work was done within 1 h, and the bats were released back into their roost to ensure that they had approximately 12 h of acclimatization time before nighttime activity. The GPS sampling time for each season was 1 h before sunset to 1 h after sunrise, 19:00–7:00 in autumn and 20:00–7:00 (UTC + 8 h) in summer, with a sampling interval of 10 min.

In this study, the mean body mass of *I. io* was 58.88 ± 6.58 g in summer and 66.67 ± 5.41 g in autumn (see Additional file 1: Table S1). Seven individuals were tracked in summer and seven in autumn; thus, a total of 14 bats were tagged with GPS loggers. The average weight of the coating and the total GPS logger was 3.5 ± 0.2 g, approximately 4.77–6.75% of the body mass of the bats. Although bats’ load weighing should not exceed 5% of their body mass in principle [39], mounting evidence shows that a ratio less than 10% may not significantly influence the normal activities of bats [40, 41]. Moreover, our preliminary field experiments also indicated that the GPS loggers did not significantly impact either the body mass or activities of *I. io* (see Additional file 1 for details).

### Insect resource survey

In order to explore whether insect resources were randomly distributed in each season at the study area, we selected five sites to survey insect resources in each season. The five sites were selected based on frequencies of spatial use by *I. io*. The first site was located in an area where *I. io* visited most frequently (areas with the highest home range overlap among individuals). The second site was located in an area where bats often visited (areas with high home overlap among individuals). The third site was in an area with low bat visitation rates (areas used by two individuals). The fourth site was situated in an area favored by an individual, where individual specialization was strong (i.e., the home range of one bat does not overlap with those of other individuals). The fifth site was located in an area where bats did not visit. Insects were collected for three nights, with the survey time fixed at 20:00–5:00 at each sampling site. We placed insect traps in areas with high vegetation cover, including forest edges, agricultural fields, and grasslands. Insect traps consisted of a 2.5-gallon polypropylene bucket insect collector, a 30-cm diameter plastic funnel with smooth interior walls, and a 1000-W high-pressure mercury lamp suspended directly above the funnel. The traps were installed at a consistent height of two meters above the ground and were firmly secured in place with a combination of nylon wire and wire suspension. The high-pressure mercury lamp was held in place with a nylon thread, and 75% alcohol was added to the polypropylene bucket for rapid dispatching of the insects. All samples collected were individually sorted and identified by entomologists to order level.

### GPS data collection and preprocessing

The GSM modules successfully transmitted GPS data, and we obtained data from fourteen bat individuals (seven in summer and seven in autumn). Then, we manually deleted any incorrect sites that were caused by the difficulty in obtaining satellite signals. We recorded GPS data for 23 nights in summer and 17 nights in autumn, with an average of 2.86 ± 0.77 nights per bat.

### Space resource utilization analysis

To characterize the use of spatial resources by *I. io*, we used the kernel density estimation (KDE) method to explore the utilization distribution (UD) of spatial use. Here, we used “95% KDE” to estimate the home ranges of individuals and populations in both seasons and set “50% KDE” as the respective core activity area [42]. For calculating the UD of the population, we pooled the data of all individuals in each season. Finally, the utilization distribution was calculated using the kernelUD function in the adehabitatHR package for R [43], with the relevant parameters set to h = “href”, grid = 200.

### Spatial specialization analysis

Considering space as a resource, we used the spatial individual specialization index (SpatIS) and the spatial individual complimentary specialization index (SpatICS) to quantify the degree of specialization in the two seasonal populations. SpatIS calculates the degree of specialization of each individual within the population, and SpatICS compares the degree of specialization of individuals in terms of spatial use with other individuals in the population [17]. We also employed the spatial overlap index, which can be used to quantify the extent of home-range overlap between individuals. By combining these two methods, we aimed to provide a more comprehensive explanation of the overlap and specialization in the horizontal space use of individuals.

The default overlap index for the calculation of SpatIS and SpatICS is a method for calculating the volume intersection (VI). SpatIS was calculated by comparing the intersection between the volume of distribution of space utilization of a single bat (UD_i_) and the volume of distribution of space utilization of the whole population (UD_pop_). SpatICS was calculated from the intersection of UD_i_ with the volume of distribution of space utilization of a population composed of individuals other than itself (UD_rest_), as follows:

$$\begin{aligned} & {\text{SpatIS}}_{{{\text{i}},{\text{pop}}}} = 1 - \int_{\Omega } {\min \left[ {{\text{UD}}_{{\text{i}}} \left( {{\text{x}},{\text{y}}} \right),\,{\text{UD}}_{{{\text{pop}}}} \left( {{\text{x}},{\text{y}}} \right)} \right]} \,{\text{dxdy}}, \\ & {\text{SpatICS}}_{{{\text{i}},{\text{rest}}}} = 1 - \int_{\Omega } {\min \left[ {{\text{UD}}_{{\text{i}}} \left( {{\text{x}},{\text{y}}} \right),\,{\text{UD}}_{{{\text{rest}}}} \left( {{\text{x}},{\text{y}}} \right)} \right]} \,{\text{dxdy}}. \\ \end{aligned}$$

where Ω represents the study area; i represents a single individual; pop represents the whole population, and rest represents the individuals in the population other than the i individuals. SpatIS and SpatICS range from 0 to 1, with higher values representing a higher degree of individual specialization and lower values representing more generalized individuals. To test the significance of SpatIS and SpatICS in *I. io*, we estimated the differences between the observed SpatIS (or SpatICS) and randomized SpatIS (or SpatICS) with a one-sided *t* test in the two seasons based on the methods followed by Kerches-Rogeri et al. [17], using a significance level α = 0.05. To conduct the test, we randomized all recorded bat spatial location data among individuals, and the procedure was repeated 1000 times. Finally, we also determined statistical power when a significant result (*p* < α) was detected by the *t* test described above.

We also employed spatial overlap indices to quantify the degree of overlap between individuals. We defined and calculated the overlap in the use of home range and core activity areas between individuals using the method followed by Fieberg et al. [44]. The Home_AOI_ index was used to represent the spatial overlap of home ranges between individuals, and between individual and population. Moreover, the Core_AOI_ index was used to represent the spatial overlap of core areas between individuals, and between individual and population. Here the method used the default overlap index to calculate the volume intersection (VI):

$$\begin{aligned} & {\text{HomeAOI}}_{{{\text{i}},{\text{j}}}} = \int\limits_{ - \infty }^{\infty } {\int\limits_{ - \infty }^{\infty } {\left[ {{\text{UD}}_{{\text{i}}} \left( {{\text{x}},{\text{y}}} \right),\,{\text{UD}}_{{\text{j}}} \left( {{\text{x}},{\text{y}}} \right)} \right]} } \,{\text{dxdy}}, \\ & {\text{CoreAOI}}_{{{\text{i}},{\text{j}}}} = \int\limits_{ - \infty }^{\infty } {\int\limits_{ - \infty }^{\infty } {\left[ {{\text{UD}}_{{{\text{i}},50}} \left( {{\text{x}},{\text{y}}} \right),\,{\text{UD}}_{{{\text{j}},50}} \left( {{\text{x}},{\text{y}}} \right)} \right]} } \,{\text{dxdy}}. \\ \end{aligned}$$

where i and j represent two independent individuals in the population. If one of i or j is the home range of the whole population, then the result of the calculation is the degree of overlap between the individual and the home range or core area of the whole population. This index can provide an overlap measure for any two home ranges or core areas. The UD overlap index ranges from 0 to 1. A value closer to 0 indicates that the two areas being compared have minimal overlap, while a value of 1 indicates that the utilization distributions (UDs) of the two entities completely overlap. Both methods were performed using the adehabitatHR package for R to calculate the overlap between UDs in SpatIS and Overlap VI with the parameters set to h = “href”, grid = 200, and meth = “VI”; we also used percent = 95 and percent = 50 to calculate the overlap between home ranges and core areas, respectively.

### Statistical analysis

Shapiro–Wilk tests were performed to test whether the data conformed to a normal distribution. All statistical analysis were performed in R 4.1.1 (R. Core Team, 2021). Based on the number of classified and counted insects, we used the Shannon index to assess the diversity of insect resources in both seasons. One-way ANOVA was used to compare the diversity of insect resources among five sampling sites in both summer and autumn. Moreover, one-way ANOVA was also used to assess differences in insect abundance among five sites in summer, while the Kruskal–Wallis test was used to compare insect abundance among the five sites in autumn, as the insect abundance data recorded at the five sites during autumn did not follow a normal distribution. Additionally, the Mann–Whitney *U* test was used to analyze differences between summer and autumn in insect abundance, home range size, and core area size at the individual level. An independent sample *t*-test was used for the differences between summer and autumn in insect diversity and the level of spatial individual specialization.

To assess the factors influencing spatial individual specialization in *I. io*, we employed a linear mixed model (LMM) using the lmer function in the lme4 R package [45]. The model used the SpatIS as the response variable, and six factors were screened as predictor variables: body mass, land use (transformed by log10), elevation, distance from the road (road distance), the density of nearby villages (village density), and insect diversity (refer Table 1 for details), with seasons and individual identity as random effects. We calculated the variance inflation factor (VIF) between the predictor variables and found that the VIF between any two predictor variables was < 5, indicating that no multicollinearity existed between the predictor variables. To validate the LMM, we utilized the DHARMa R package developed by Hartig [46], generating residuals using the simulateResiduals function with 1000 simulations. We assessed residual dispersion with the testDispersion function and confirmed normal distribution through the use of the testUniformity function. Additionally, we also selected optimized linear models using dredge and model.avg function in the package ‘MuMIn’ [47] using the same variables to LMM. During the model selection, we employed Akaike's information criterion corrected for small sample size (AICc) was used to compare the strengths and weaknesses of the fitted models. We calculated the Akaike weights (*w*_*i*_) for each model to assess the relative likelihood of a given model and to compare it with the other candidate models. If the difference in AICc between the second model and the first best-fit model was > 2 (ΔAICc > 2), then the first model was considered as the best-fit model. If the ΔAICc was ≤ 2, model averaging was performed using the model.avg function to obtain the predictor variables that best explained the variation in SpatIS.

**Table 1:** Description of the predictor variables involved in this study that may affect the spatial individual specialization index of *Ia io*

Variables	Description
Body mass	Weight per bat (g)
Land use	For different land use types corresponding to different land use type indices, bare land = 1, woodland and scrub = 2, grassland, farmland = 3, water bodies = 4
Elevation	Local altitude (m a.s.l.) corresponding to each bat activity location
Road distance	Distance from the road, divided into four categories (< 10, 10–100, 100–500 and > 500) converted into weights, i.e., 1, 0.5, 0.25 and 0 respectively
Village density	The density of villages within the home range of each bat (No. villages km^−2^)
Insect diversity	Diversity index of insects (Shannon diversity)

## Results

### Distribution patterns and interseasonal variation of insect resources

**Fig. 2:**
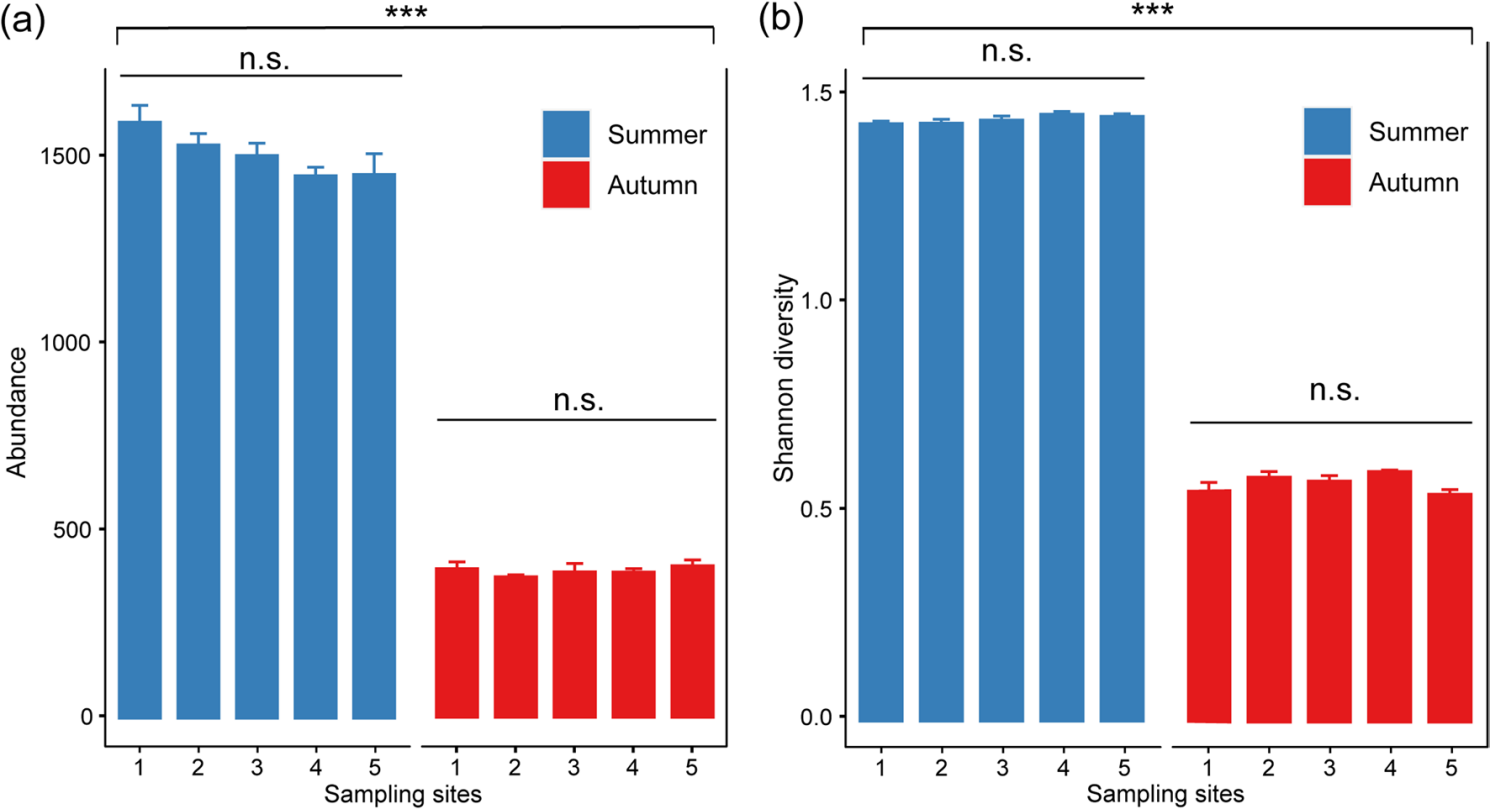
Insect abundance and diversity among five sampling sites and seasons. **a** The abundance of insects, **b** the Shannon diversity of insects (n.s indicates no significant differences in data, i.e., *P* > 0.05, ***P < 0.001)

Using insect traps, we collected 22,745 and 5871 insects from five locations in summer and autumn, respectively. These insects were classified into 12 orders: Blattaria, Coleoptera, Diptera, Hemiptera, Hymenoptera, Lepidoptera, Neuroptera, Odonata, Orthoptera, Trichoptera, Ephemeroptera, and Mantodea. The first ten orders have been confirmed in bat diets [36]. We found that there was no significant difference in insect abundance among the five sites in either summer (*F* = 2.333, *P* = 0.126; Fig. 2a) or autumn (*H* = 3.367, *P* = 0.498; Fig. 2a). Similar patterns were also observed in insect diversity in both summer (*F* = 1.567, *P* = 0.257; Fig. 2b) and autumn (*F* = 2.878, *P* = 0.080; Fig. 2b). However, the insect abundance in summer was significantly higher than that in autumn (*W* = 22.774, *P* < 0.001; Fig. 2a). Moreover, we found that insect diversity was significantly higher in summer than in autumn (*t* = 104.44, *P* < 0.001; Fig. 2b).

### Home range and core area sizes of *I. io* at population and individual levels

**Fig. 3:**
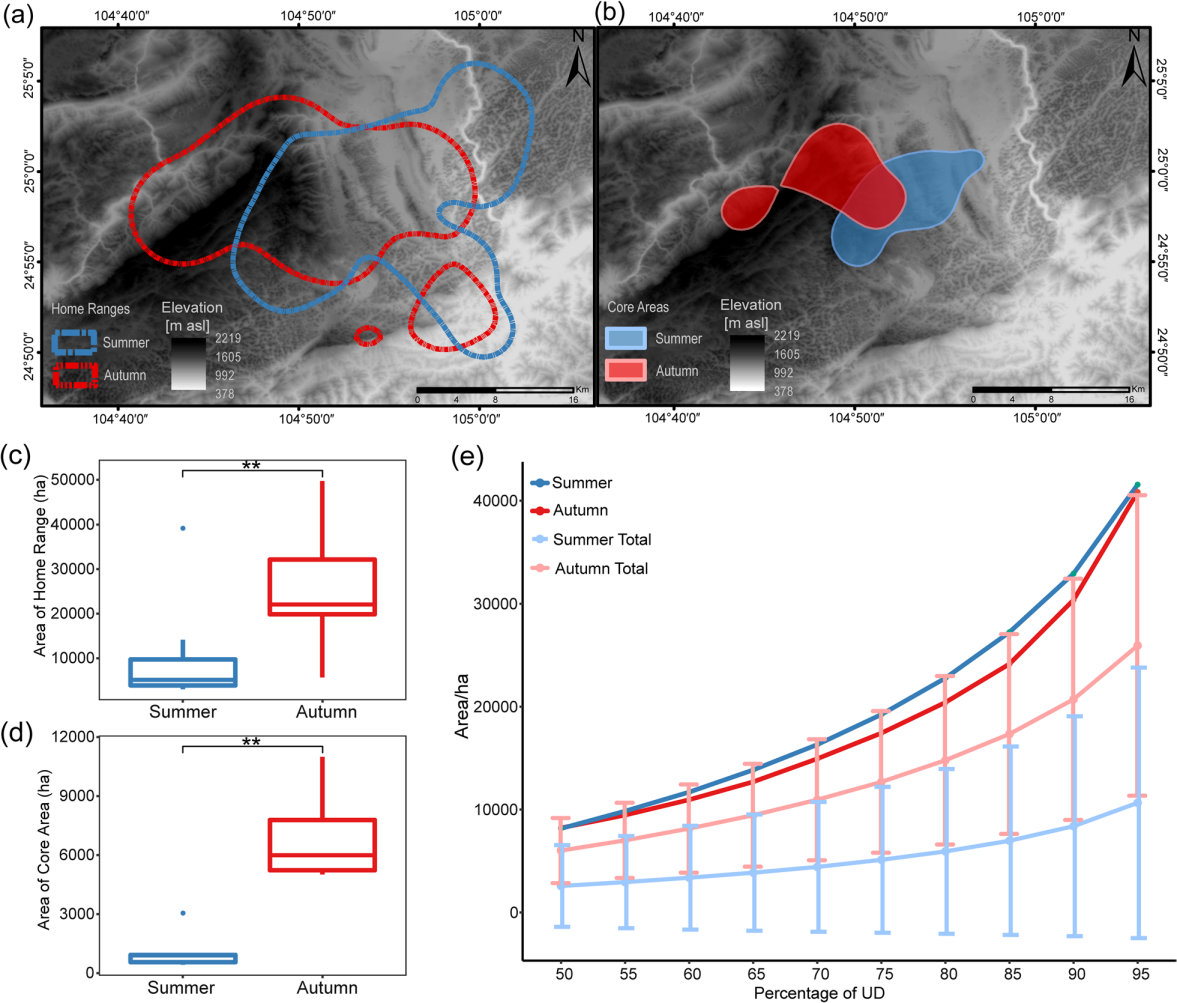
Spatial resource utilization of *I. io* in summer and autumn. **a** Home range utilization distribution of the population, with dotted colors representing different seasons. **b** Core area utilization distribution of the population, with different colored facets representing different seasons. **c** Differences in home range of individuals between seasons. **d** Differences in core areas of individuals between seasons. **e** Trends in different spatial utilization distribution (UD) of individuals and seasonal populations, the summer total and the autumn total represent the overall space utilization of these populations and the bars represent the standard deviation of the individual-level space utilization for the summer and autumn populations

The home range and core area sizes of the *I. io* population in both seasons were basically the same. The home range sizes were 41,557.20 ha and 40,837.38 ha in summer and autumn, and the core area sizes of the entire population were 8157.52 ha and 8217.28 ha, respectively (Fig. 3a, b, and e). The average home range per bat was 10,652.85 ± 13,141.99 ha (range 3033.50–39,185.26 ha) in summer and 25,938.43 ± 14,594.78 ha (5681.74–49,744.09 ha) in autumn and was significantly greater in autumn than in summer (*W* = 43, *P* < 0.001; Fig. 3c). The core area per bat ranged from 424.82 to 11,337.87 ha (mean ± SD = 2571.60 ± 3967.64) in summer and from 734.52 to 10,996.70 ha (mean ± SD = 6013.64 ± 3164.67) in autumn and was also observed to be significantly larger in autumn than in summer (*W* = 38, *P* < 0.001; Fig. 3d). As the utilization distribution (UD) percentage increased, the size of spatial resource utilization of the summer and autumn populations remained relatively consistent. However, there was a noticeable difference in the spatial range of individual-level utilization (Fig. 3e).

### Spatial individual specialization of *I. io*

**Fig. 4:**
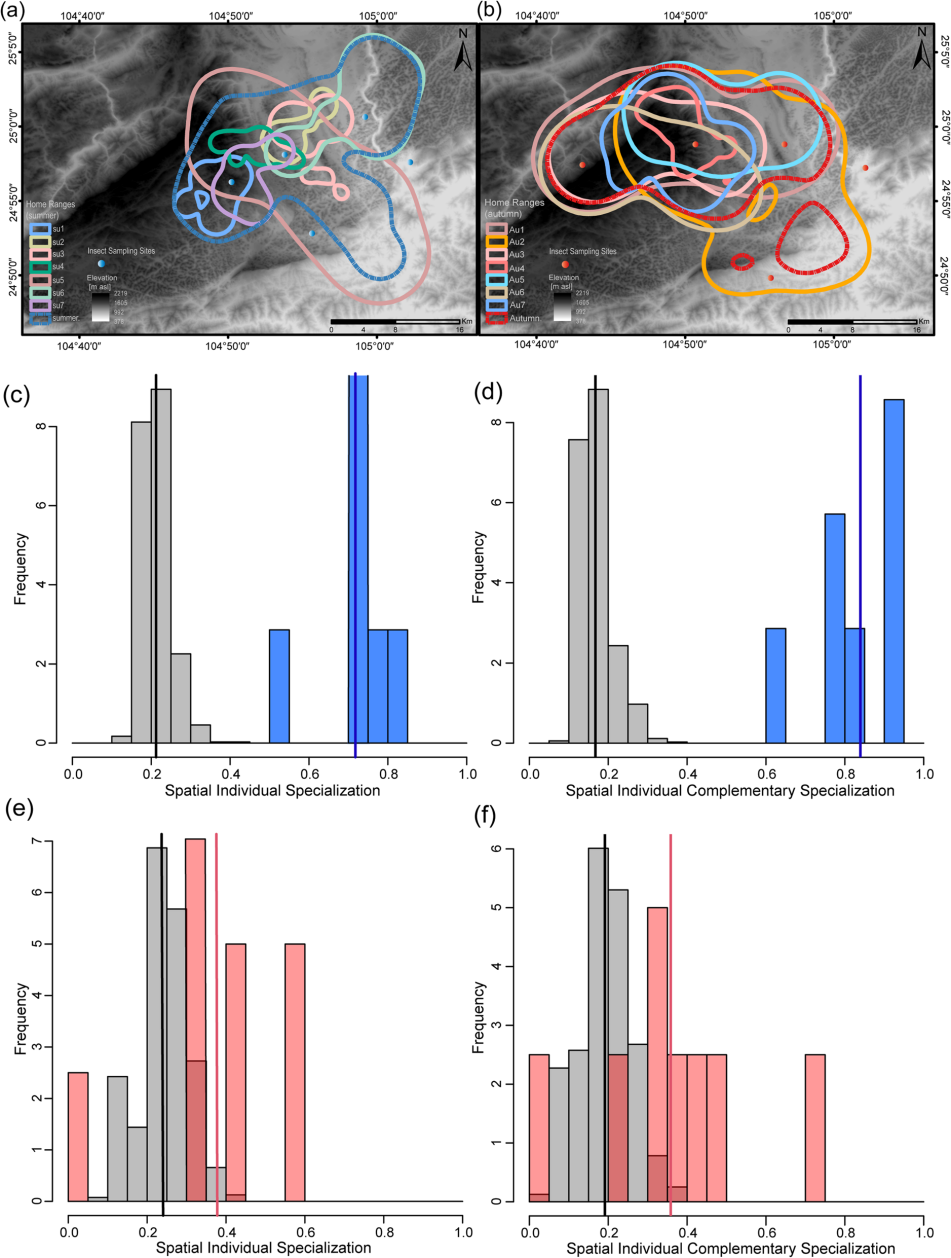
Utilization distribution of home range (95% KDE) of *I io* individuals and the insect sampling sites **a** in summer and **b** in autumn. The different individuals are indicated by different colored outlines. **c** Spatial individual specialization index (SpatIS) for summer individuals. **d** Spatial individual complementary specialization index (SpatICS) for summer individuals. **e** SpatIS for autumn individuals. **f** SpatICS for autumn individuals. In **c**–**f** the blue bars represent observed values of SpatIS and SpatICS in summer, the pink bars represent observed values of SpatIS and SpatICS in autumn; the blue and red lines represent the mean of the respective observed values; the gray bars represent values in SpatIS and/or SpatICS after being randomized, black line represents mean values after randomization

The spatial use of *I. io* individuals displayed a degree of overlap in both summer and autumn, but the overlap was greater in autumn (Fig. 4a, b). In summer, the SpatIS of I. io was 0.718 (compared to 0.212 after location randomization; *t* = 14.10, *df* = 7, *P* < 0.001, *power* = 1; Fig. 4c), and the SpatICS was 0.839 (compared to 0.167 after location randomization; *t* = 16.13, *df* = 7, *P* < 0.001, *power* = 1; Fig. 4d). In autumn, the SpatIS was 0.378 (compared to 0.241 after location randomization; *t* = 2.31, *df* = 7, *P* < 0.05, *power* = 1; Fig. 4e), and the SpatICS was 0.358 (compared to 0.191 after location randomization; *t* = 2.27, *df* = 7, *P* < 0.05, *power* = 1; Fig. 4f). Overall, the results indicated that spatial individual specialization existed in both seasons. Moreover, both SpatIS and SpatICS showed that *I. io* in summer had a higher level of individual specialization and less spatial overlap, while *I. io* in autumn had a lower level of spatial specialization and greater spatial overlap.

**Fig. 5:**
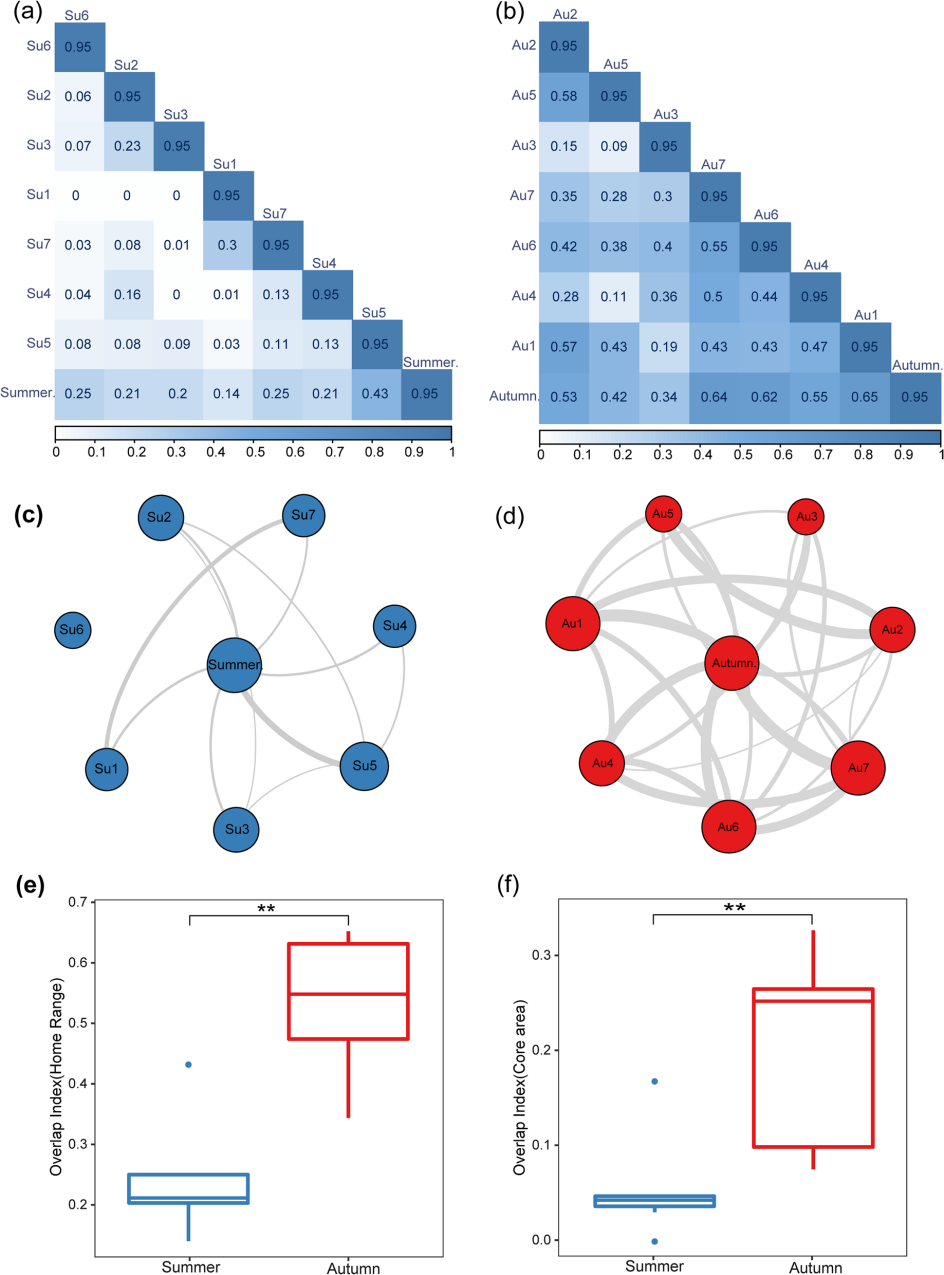
Spatial use overlap of *I. io* in summer and autumn. Heat map showing **a** the overlap index among per bat home range in summer and **b** in autumn. The darker blue represents a larger overlap (ranging from 0 to 0.95 with progressively deeper colors). Core area overlap network with individual bats as nodes in summer (**c**) and in autumn (**d**). The widths of the links represent the overlap between individuals, with thicker representing greater overlap. **e** Differences in home range overlap index of individuals between seasons. **f** Differences in core area overlap index of individuals between seasons

The home range values between individuals showed smaller overlap in summer (Fig. 5a) but larger overlap in autumn (Fig. 5b). Using the overlapping network map of the core areas, we found that the overlap between individuals was greater in autumn compared to summer, showing a more complex network structure and thicker connections (Fig. 5c, d). Additionally, the overlap indices of home range (*W* = 47, *P* < 0.01; Fig. 5e) and core area utilization (*W* = 46, *P* < 0.01; Fig. 4f) between *I. io* individuals were significantly greater in autumn than in summer.

### Factors influencing the spatial individual specialization of *I. io*

The analysis of the LMM showed that insect diversity was the only significant predictor of individual specialization in bats (Table 2). And the residuals of the LMM were normally distributed (see Additional file 1: Fig. S1), which suggests the LMM is valid. Additionally, our analysis indicated that the best model of individual specialization variation included only insect diversity as a predictor variable. The models were ranked from best to worst according to the AICc values, and the difference in AICc between the second model and the first model was greater than 2 (ΔAICc > 2), indicating that the first model was the best-fit model (see Additional file 1: Table S2). In sum, these results showed that the higher the diversity of insect resources available to individuals of *I. io*, the larger the SpatIS index (i.e., the higher the degree of individual specialization).

**Table 2:** Linear mixed models assessing the influence of body mass, land use, elevation, road distance, village density and insect diversity on spatial individual specialization (SpatIS) of *I. io*

Predictive variables	Estimate	SE	df	t	*P* value
(Intercept)	1.627	0.554	14	2.938	**0.011**
Body mass	− 0.007	0.004	14	− 1.998	0.066
Land use	− 0.180	0.088	14	− 2.056	0.059
Elevation	0.000	0.001	14	0.371	0.716
Road distance	− 0.002	0.004	14	− 0.341	0.738
Village density	− 0.049	0.056	14	− 0.866	0.401
Insect diversity	0.192	0.066	14	2.887	**0.012**

Statistically significant variables are shown in bold

## Discussion

Our results showed that the spatial niche breadth (population home range and core area) of *I. io* did not increase in autumn when insect resources were reduced, rather being similar in summer and autumn, in contrast to our first hypothesis. Since the first hypothesis did not hold, our results thus did not support our second hypothesis. However, we found that *I. io* showed individual specialization in space use in both summer and autumn, with a higher degree of spatial individual specialization in summer and an increase in individual niche breadth and a decrease in the degree of individual specialization in autumn. These results supported our third hypothesis that the spatial niche breadth and spatial individual specialization would vary among individuals of the same population across seasons. Additionally, we found that insect diversity was the main factor influencing the degree of spatial individual specialization, supporting our final hypothesis. This study is the first to explore changes in individual and population spatial niche utilization patterns within the same population across seasons and to determine the factors influencing spatial individual specialization.

Researchers often use the term “jack-of-all-trades, master-of-none” to explain why species do not expand their niches indefinitely by using all available resources [48]. A species occupying a wide range of resources will suffer higher costs of adaptation, and thus trade-offs are made in niche expansion [49]. Indeed, trade-offs in niche breadth have been reported in many dimensions. For example, specialized species of herbivores can quickly detect and disrupt plant-specific defense mechanisms, yet this may come at the cost of a narrower niche (a limited range of available plants) [50]. In terms of foraging, if an individual increases the efficiency of consuming a given resource type, it will lose the ability to consume other resource types [51], and these trade-offs can also be reflected in spatial movement patterns [52]. In this study, we found that there were no significant differences in insect abundance or diversity among the five sites in both summer and autumn, suggesting that insect resources may be distributed randomly within the study area in each season (Fig. 2a, b). However, decreases in insect abundance and diversity were observed from summer to autumn (Fig. 2a, b), but home range and core area sizes of the *I. io* population did not increase in autumn when insect resources were scarce. The results suggested that the spatial niche breadth of *I. io* at the population level was similar in summer and autumn (Fig. 3a, b, and e). Our results were not consistent with some previous findings showing that vertebrate populations expand their home ranges when available food resources are decreased [18–21]. Here we discuss two possible explanations for the stable space use of the population across seasons. The first mechanism involves energy constraints during flight. An increase in the spatial niche breadth of the population would require bats to devote more energy to flying and searching for food over a larger area. However, the metabolic cost of flight is very high for bats, about 3–5 times that of other mammals with similar body size [53, 54]. In this case, we suggest that *I. io* may have used the maximum home range in both summer (41,557.20 ha) and autumn (40,837.38 ha). Thus, it would be impossible for *I. io* to expand the home range due to energy limitations despite the decrease in food resources. Second, previous studies have shown that *I. io* prey upon nocturnally migrating passerine birds in addition to insects in autumn [33, 34]. The passerine birds normally have larger body sizes and higher nutritional value than insects [55]. It seems unnecessary for *I. io* to expand the home range to search for food as insect resources decrease in autumn, since one bird may be equivalent to many insects.

As mentioned above, the spatial niche breadth of *I. io* at the population level did not increase in autumn when food became limiting. In this case, our results did not support the predictions of OFT or the NVH. It is believed that exploring the niche variation of individuals within a population can more accurately detect changes in the population niche breadth [2, 51]. Here, the individual niche breadth of space use in *I. io* was greater in autumn than in summer (Figs. 4a, b and Fig. 5e, f). That is, generalist strategies should be favored in autumn when insect resources decrease. In this case, the population niche breadth of space use in *I. io* would in theory increase, and this was not the case in our study. This may be because increases in individual niche breadth were offset by the decreases in between-individual variation (spatial individual specialization), resulting in a dynamically stable population niche breadth for space use between the two seasons. These results suggested that the niche breadth of a population (or species) may be regulated by a combination of individual niche breadth and between-individual variation [12].

Individual specialization is a common phenomenon in natural populations, and it has been studied extensively. In seabirds and bats with high mobility and dispersal abilities, individual specialization can reduce competition between individuals and provide advantages in reproductive performance and foraging [17, 28, 56]. In this study, to strengthen the association between individual specialization and spatial resource utilization, we used both the classical spatial overlap index and the latest individual specialization index to quantify the level of spatial individual specialization, and both methods obtained consistent results. We found that spatial individual specialization existed in both seasons, and *I. io* in summer had a higher level than in autumn (Fig. 4). Moreover, spatial use overlap analysis showed that *I. io* displayed a larger overlap in autumn than in summer, also supporting the results of spatial individual specialization (Fig. 4). These results present strong evidence that spatial individual specialization in *I. io* existed and varied between individuals of the same population in response to insect resource variation across seasons. As predicted by Svanback and Bolnick, individual specialization can promote population differentiation and species coexistence [57, 58]. In summer, specialist strategies of space use in *I. io* should be selected to avoid intraspecific competition, because insect resources are abundant and diverse. In this case, every individual may forage in a different subarea with low overlap. Thus, spatial individual specialization may improve the efficiency of individual utilization of resources in a specific space and provide a key mechanism for *I. io* to survive in summer. In autumn, individual niche breadth was increased by decreasing spatial individual specialization to use a larger area to compensate for the lower insect resource availability. Although nocturnally migrating birds can be hunted by *I. io* as a predictable food resource in autumn, our previous study showed that *I. io* may prey on birds at higher flight altitudes in autumn rather than preying on insects as in summer [33]. Thus, predation on birds by *I. io* may not expand only their two-dimensional home range. In summary, whether to use a wider but more competitive resource distribution area or to reduce competition within their respective small areas, *I. io* must make a trade-off to find the optimal space use strategy. The plasticity of animal foraging strategies is an adaptive response to environmental change that can improve individual fitness by maximizing energy intake and reducing intraspecific competition [57].

Spatial individual specialization may be influenced by environmental and resource changes as well as phenotypic and personality differences among individuals [17, 29, 59]. Our results suggested that insect diversity determines the level of specialization of *I. io* in spatial resource use, while other variables (i.e., environmental variables and individual body mass) did not explain the variation in spatial individual specialization. Here we discuss two possible explanations for the results concerning the factors impacting on spatial individual specialization. First, landscape variation affects the distribution and diversity of food resources, and individual differences (e.g., body mass) affect the utilization of their own food resources. Thus, it may be sensible that body size and landscape variables (land use, road distance, village density, elevation) may not directly influence spatial individual specialization in *I. io*. In addition, food resource changes affect the level of intraspecific competition, one of the main drivers of between-individual variation [51]. Optimal foraging theory predicts that intraspecific competition can reduce individual specialization depending on the ranking patterns of preference for food. That is, individuals may initially prefer different resources, and a decrease in resources would increase the intensity of competition, causing them to concentrate on shared secondary resources and thereby reducing the level of individual specialization [12, 60]. Moreover, individual specialization may even disappear when food resource availability decreases, i.e., individuals may adopt generalist strategies [58]. However, not all species can utilize only a fixed or a single food resource, and individual specialization may occur when ecological opportunities are available [48]. For example, populations that use both marine and terrestrial resources show stronger individual specialization than populations that use only one of these resources [12]. In this study, although *I. io* showed individual specialization in autumn when insect resources were scarce, the bats comprised a generalist population and exhibited low levels of individual spatial specialization. The scarcity of food resources increased intraspecific competition and decreased individual specialization. However, specialization did not disappear completely; this may have been due to the emergence of nocturnally migrating passerine birds as an ecological opportunity to facilitate individual specialization. Alternatively, maintaining a certain degree of spatial individual specialization may have a beneficial effect on reducing intraspecific competition within the *I. io* population. Specifically, by avoiding the complete loss of spatial specialization, the population may be able to optimize resource utilization and minimize potential conflicts among individuals. These results suggested that intraspecific competition and ecological opportunity are mediated by food resource changes that affect spatial individual specialization, and the observed pattern of change may also be influenced by individual preferences for specific spatial resource ranks, which could be consistent with dietary specialization. However, the relationship between spatial specialization and dietary specialization should be a focus of future studies.

We acknowledge that our study has several limitations. One limitation is that the dietary niche breadth at the population and individual levels of *I. io* has not been simultaneously investigated, a situation that makes it difficult to determine whether all individuals in both seasons would consume all of the insects that were surveyed in our background resource survey. Future studies should investigate the relationships between dietary and spatial niche dimensions at the individual level. Second, it was very difficult to survey the diversity and abundance of nocturnally migrating birds in the study area, and thus the effects of bird resource on spatial use of *I. io* remain unclear, especially for the three-dimensional home range. The last is the study’s relatively small sample size, as we obtained spatial use data from only seven individuals in each season. However, this may not affect the reliability of our conclusion for four reasons. First, the population size of *I. io* is about 80, which is very small, and thus the fourteen individuals may be representative. Second, it is generally believed that under natural conditions in the wild, individual movement and foraging patterns are independent [37, 38]. Thus, the individual foraging movement patterns may be independent because of the small population size and large home range in *I. io*. Third, the fourteen individuals also were independent of each other because we have marked most individuals in the population since 2016. Fourth, the GPS tracking experiments in each season were performed within at least 1 month, a factor that reduced the probability that individuals caught at the same time would display similar movement patterns. In this study there were only males in the study area, and thus our tracked samples were limited to male bats. Although this may not be sufficient to fully explore the ecological niche of the entire population, our results can reflect the spatial niche variation in the male colony across seasons. In future studies it will be important to include data on both sexes in order to better understand the population niches of the species and facilitate the verification of our study's findings. The final limitation is that it was very difficult to track the same individuals across seasons due to the small population size and low recapture probability, and thus it was not possible to obtain spatial use data for the same group of individuals in different seasons. As such, the possibility that the changes in spatial use witnessed between seasons may be ascribed to individual-specific characteristics (e.g., different personalities, reproductive status) rather than seasonal fluctuations cannot be ruled out. However, we believe that our data from seven individuals in each season can partly reflect the trends in spatial niche breadth at both the population and individual levels. Moreover, individual niche breadth of six individuals in autumn was increased compared to summer, a result that supports our above view. Future research will aim to track the same individuals across seasons, as this will enable a better understanding of the relationship between population spatial niche changes, individual niches, and specialization caused by seasonal variation.

## Conclusions

Herein, we investigated linking changes in the spatial use of individuals and the population in *I. io* across seasons. The results showed that the spatial niche breadth of the *I. io* population was similar in summer and autumn despite insect resources being reduced in autumn. Moreover, we found that spatial individual specialization existed but varied across seasons in response to variation in insect resources. In summer, specialist strategies of space use in *I. io* were likely favored to avoid intraspecific competition when insect resources were abundant. In autumn, individuals expanded their niche breath, selecting a more generalist strategy and thereby reducing individual specialization. This behavior can lead to an improvement in their overall fitness, as it reduces competition among individuals. These results suggest that the spatial niche breadth of a population (or species) may be regulated by a combination of individual niche breadth and spatial individual specialization. Although studies on individual specialization initially focused on diet, our work suggests that space can also be considered as resource to investigate the evolution of niche breadth, and thus the present study provides new insights into niche theory.

## Supplementary Information

**Additional file 1: Table S1** The basic information for each individual bat in this study. **Table S2.** Model selection based on the AICc assessing the influence of body mass, land use, elevation, road distance, village density, and insect diversity on spatial individual specializationof *Ia io*. Models were ranked according to AICc values from lowest to highest. The signs of the regression coefficients of the predictor variables are shown in parentheses.

## Data Availability

Data are available from Figshare at https://doi.org/10.6084/m9.figshare.21717086.v3.

## Abbreviation

GPS: Global positioning system
