# Supplementary material for: Linking changes in individual specialization and population niche of space use across seasons in the great evening bat (Ia io)
Source: Mov Ecol. 2023 Jun 7;11:32. doi: 10.1186/s40462-023-00394-1 (PMC10249169; doi:10.1186/s40462-023-00394-1)
Supplement: Supplementary file 1 — Additional file 1: Table S1 The basic information for each individual bat in this study. Table S2. Model selection based on the AICc assessing the influence of body mass, land use, elevation, road distance, village density, and insect diversity on spatial individual specializationof Ia io. Models were ranked according to AICc values from lowest to highest. The signs of the regression coefficients of the predictor variables are shown in parentheses. [file 40462_2023_394_MOESM1_ESM.docx]

## Additional file of “Linking changes in individual specialization and population niche of space use across seasons in the great evening bat (*Ia io*).”

Zhiqiang Wang, Lixin Gong, Zhenglanyi Huang, Yang Geng, Wenjun Zhang, Man Si, Hui Wu, Jiang Feng and Tinglei Jiang

**Appendix S1**

**Table S1.** The basic information for each bat.

| Bat | Season | Sex | Forearm length(mm) | Body mass(g) |
| --- | --- | --- | --- | --- |
| Su1 | Summer | Male | 74.14 | 52.47 |
| Su2 | Summer | Male | 74.63 | 53.6 |
| Su3 | Summer | Male | 77.47 | 51.81 |
| Su4 | Summer | Male | 76.85 | 60.37 |
| Su5 | Summer | Male | 75.12 | 57.21 |
| Su6 | Summer | Male | 76.93 | 68.84 |
| Su7 | Summer | Male | 78.78 | 67.83 |
| Au1 | Autumn | Male | 76.71 | 68.29 |
| Au2 | Autumn | Male | 79.12 | 67.91 |
| Au3 | Autumn | Male | 76.66 | 54.38 |
| Au4 | Autumn | Male | 74.54 | 73.45 |
| Au5 | Autumn | Male | 72.91 | 67.72 |
| Au6 | Autumn | Male | 78.36 | 66.54 |
| Au7 | Autumn | Male | 72.26 | 68.38 |

**Table S2.** Model selection based on the AICc assessing the influence of body mass, land use, elevation, road distance, village density, and insect diversity on spatial individual specialization (SpatIS) of I io. Models were ranked according to AICc values from lowest to highest. The signs of the regression coefficients of the predictor variables are in parentheses.

| Model | Predictive variables | *df* | LogL | AIC | ΔAIC | *w_i_* |
| --- | --- | --- | --- | --- | --- | --- |
| 1 | Insect diversity(+) | 5 | 12.66 | −7.82 | 0.00 | 0.43 |
| 2 | (Null) | 4 | 8.97 | −5.51 | 2.32 | 0.13 |
| 3 | Insect diversity(+), land use(-) | 6 | 14.35 | −4.71 | 3.12 | 0.09 |
| 4 | Land use(−) | 5 | 11.02 | −4.54 | 2.29 | 0.08 |
| 5 | Body mass(−), insect diversity(+) | 6 | 13.98 | −3.95 | 3.87 | 0.06 |
| 6 | Body mass(−) | 5 | 10.53 | −3.56 | 4.26 | 0.05 |
| 7 | Insect diversity(+), road distance(+) | 6 | 13.13 | −2.26 | 5.57 | 0.03 |
| 8 | Insect diversity(+), village density(+) | 6 | 13.02 | −2.04 | 5.79 | 0.02 |
| 9 | Elevation(+),insect diversity(+) | 6 | 12.93 | −1.87 | 5.96 | 0.02 |
| 10 | Body mass(−),land use(−) | 6 | 12.91 | −1.82 | 6.01 | 0.02 |

('−': negative correlation; '+': positive correlation). LogL: log-likelihood value.


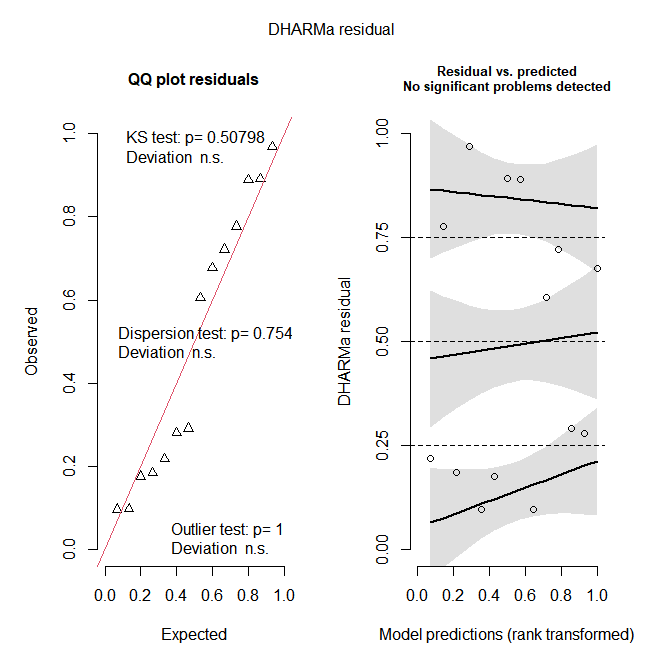


**Figure S1.** The distribution pattern of the residuals of the LMM model. The left panel is a QQ-plot used to detect overall deviations from the expected distribution, with tests for correct distribution (KS test), dispersion, and outliers. The right panel shows the residuals against the predicted values and a quantile regression with 0.25, 0.50, and 0.75 quantile lines across the plot.

**Analyzing the effect of the extra-loading on the bats:**

Although the 10% additional load on bats in the field has been shown in many experiments to have no effect on the overall behavior of bats, we have conducted an additional experiment to determine the effects of extra load on bat activity and body mass. In 2019, we prepared a replacement similar to the weight of the GPS, using a marker that is about the same size and weight as the GPS tag (the substitute is made of alloy) and a metal antenna of the same length as the GPS tag. Although we have continuously tagged 25 *I. io* bats for three months, we have only recaptured five tagged bats. Moreover, although we have recaptured some other tagged individuals, the markers have fallen off. The time between the five bats from wearing the tag to being recaptured was 2-5 days, and we compared the body mass and physical condition of these bats with other *I. io* individuals captured on the same day. First of all, we compared the body mass of the five bats when they were captured and equipped with tags to their mass when recaptured a few days later for tag removal. Because the body mass of bats varies in a day, so we caught them in the early hours of the morning. Using a paired-sample Wilcoxon test, we found no significant changes in body mass before and after wearing the tags (*P* = 0.44). Then we examined the physical condition of each bat, and we found that four of the five bats we tagged showed signs of bird catching (from traces of blood in the tail or mouth, and feathers in the claws), suggesting that the *I. io* continued to engage in high-altitude bird hunting after wearing the tags, which may have little effect on their maneuverability.
